# Multiplexed characterization of cancer immune response in FFPE biopsies - initial performance of a new approach

**DOI:** 10.1186/2051-1426-1-S1-P54

**Published:** 2013-11-07

**Authors:** Chichung Wang, Rebecca King, Michael Feldman, Clifford Hoyt

**Affiliations:** 1 Perkin Elmer, Inc., Hopkinton, MA, USA; 2 CHOP, Philadelphia, PA, USA; 3 Hospital of UPENN, Philadelphia, PA, USA

## Background

Recent successes with experimental PD-L1 and PD-1 drugs and with adoptive immunotherapy point to the tremendous potential clinical value of harnessing our immune systems to fight cancer. Assessing host interaction with malignant cells is most effectively achieved by phenotyping cell types in-situ in intact tissue sections. Spatial relationships and phenotype patterns can potentially predict response to immunotherapies and monitor efficacy. However, methods to perform such analyses have proven elusive due to challenges of multicolor labeling and image analysis. We report progress toward an integrated approach, combining new multiplexed labeling and imaging-based analysis, to capture expression and contextual information and reduce data to actionable metrics. In this study, we demonstrate the capability on control samples (tonsil) and a cohort of clinical breast cancer samples, by comparing automated and visual assessments.

## Methods

De-identified, excess clinical samples, tonsil and four breast cancer biopsies, were labeled for CD4, CD8, CD20, cytokeratin, and DAPI with a new serial same-species fluorescence labeling approach utilizing tyramide signal amplification (TSA), and microwave-based antigen retrieval and antibody stripping. Labels are added sequentially, each amplified independently, to achieve specific, robust, balanced labeling. Samples were imaged on the Vectra multispectral system, and analyzed with inForm pattern recognition software, which segments tissue into tumor and stroma and phenotypes cells as epithelial, killer T-cells, helper T-cells, and B-cells, or other, producing cell phenotype maps retaining spatial arrangements. Imagery was then carefully assessed by a pathologist for segmentation and cell phenotyping accuracy, and for distinguishing inter- versus intra-epithelial cells.

## Results

Labeling of tonsil samples demonstrate specificity and sensitivity estimated by a pathologist to be greater than 95%. On breast cancer samples, comparable accuracy was achieved except for areas of necrosis where it was somewhat less. More importantly, in breast cancer samples, distinguishing inter- and intra-epithelial immune cells was estimated to be nearly perfect, despite significant inflammation in non-epithelial areas immediately adjacent to tumor, in some samples.

## Conclusions

The approach shows reliable detection of phenotypes and accurate segmentation of tumor and stroma regions, to accurately assess interaction between immune cells and tumor mass, such as infiltration. We believe results support the feasibility of a practical and viable clinical workflow, in which immune response assessment is automated by computer and results are reviewed by pathologists to assure data quality.

